# Prognostic Value of a Serological-Based Clinical Model for Gastric Cancer Patients

**DOI:** 10.3390/jcm14124043

**Published:** 2025-06-07

**Authors:** Hai-Huan Feng, Wei-Han Zhang, Kai Liu, Xiao-Long Chen, Lin-Yong Zhao, Xin-Zu Chen, Kun Yang, Jian-Kun Hu

**Affiliations:** 1 Chinese Evidence-Based Medicine Center, West China Hospital, Sichuan University, Chengdu 610041, China; fenghaihuan@wchscu.edu.cn; 2 Gastric Cancer Center, Laboratory of Gastric Cancer, Department of General Surgery, State Key Laboratory of Biotherapy and Cancer Center, West China Hospital, Sichuan University, Chengdu 610041, China; weihanzhang@scu.edu.cn (W.-H.Z.); liukgcc@wchscu.edu.cn (K.L.); chenxiaolong@wchscu.edu.cn (X.-L.C.); 153795352@scu.edu.cn (L.-Y.Z.); chenxinzu@scu.edu.cn (X.-Z.C.); kunyang@scu.edu.cn (K.Y.); 3 Medical Insurance Office, West China Hospital, Sichuan University, Chengdu 610041, China

**Keywords:** gastric cancer, serological indicators, prediction model, prognosis

## Abstract

**Background**: Surgery remains the cornerstone of diagnosis and treatment for gastric cancer. This study aims to develop and validate a serology-based clinical scoring system to predict and evaluate the prognosis of gastric cancer patients. **Methods**: Clinicopathological data of primary gastric cancer patients who underwent surgical treatment from 2009 to 2018 were collected and divided into training and validation cohorts. Preoperative serological indicators were screened, and a serum risk score (SerScore) was developed using LASSO-Cox analysis. Prognosis prediction models incorporating the SerScore were established and validated. **Results**: A total of 5493 patients were screened, and 43 serological indicators were assessed. Twelve serological indicators were selected to construct the SerScore. Patients with a SerScore below the cut-off value of −1.73 had significantly better survival rates compared to those with higher scores. Multivariate Cox analysis identified SerScore, age, tumor location, T stage, and N stage as independent prognostic factors for overall survival in the training cohort. A multivariate nomogram was developed, achieving a C-index of 0.745 in the training cohort and 0.750 in the validation cohort. The nomogram demonstrated superior predictive accuracy compared to the SerScore alone, with AUC values of 0.783 versus 0.639 in the training cohort and 0.805 versus 0.657 in the validation cohort. Calibration curves closely aligned with ideal predictions in both cohorts. **Conclusions**: The SerScore model provides an effective tool for prognostic assessment in primary gastric cancer patients. This model not only enhances prognostic evaluation but also establishes a foundation for developing advanced prediction tools for gastric cancer.

## 1. Introduction

Gastric cancer is one of the most prevalent malignant tumors of the digestive system, with approximately 1 million new cases diagnosed globally each year. It ranks fifth in morbidity and fourth in mortality among malignant tumors [1]. Serum tumor markers have long been recognized as valuable tools for diagnosing and prognosticating malignant tumors [2,3,4]. Common markers such as carbohydrate antigen 19-9 (CA 19-9), carcinoma embryonic antigen (CEA), carbohydrate antigen 72-4 (CA 72-4), and carbohydrate antigen 12-5 (CA 12-5) are frequently employed to predict prognosis in gastric cancer patients. However, these markers are limited by their sensitivity and specificity, and no gastric cancer-specific serum tumor markers have been identified to date [5,6,7,8].

In recent years, advances in next-generation sequencing technology have facilitated the molecular characterization of tumors and circulating tumor DNA, enabling early diagnosis, drug sensitivity testing, recurrence monitoring, and prognosis prediction [9,10,11]. Despite these advantages, the high costs and technical complexity of sequencing currently preclude its widespread clinical application and necessitate further validation. Although individual serological indicators are associated with the prognosis of gastric cancer, prior studies have not systematically integrated all preoperative serological data. Comprehensive analysis of pretreatment baseline serological indicators is critical for identifying key prognostic factors and developing robust prognostic prediction models for gastric cancer patients.

The progression of gastric cancer affects various physiological states, including immune, nutritional, and metabolic functions that are reflected by changes in several serological indicators. For example, tumor growth increases the risk of rupture and bleeding, leading to decreased hemoglobin levels, which are strongly associated with tumor stage and survival outcomes [12]. Previous studies have explored non-tumor serological markers such as the hemoglobin, albumin, lymphocyte, and platelet (HALP) score; controlling nutritional status (CONUT) score; prognostic nutritional index (PNI); fibrinogen-to-platelet ratio (FPR); and systemic inflammation response index (SIRI) [13,14,15,16,17]. However, these investigations have been limited by their focus on specific markers, leaving a gap in understanding the prognostic value of integrating all preoperative serological indicators.

To address this gap, a comprehensive analysis of preoperative serological indicators is necessary to establish an effective systematic prognostic model for gastric cancer. Such a model would enhance the ability to predict patient outcomes and guide personalized treatment strategies. We propose that preoperative baseline serological indicators, routinely obtained during standard clinical examinations, represent a valuable and readily accessible resource for prognostic assessment in gastric cancer patients. Leveraging data from routine preoperative blood tests, biochemical blood tests, and coagulation profiles collected from a large, continuous cohort at a single center, we aim to develop and validate a comprehensive serology-based scoring system. This scoring system, integrated into a robust prediction model, has the potential to significantly enhance the accuracy of survival outcome predictions for gastric cancer patients. Ultimately, this approach is expected to contribute to improved clinical decision-making and personalized patient care.

## 2. Materials and Methods

### 2.1. Ethical Standards

This retrospective study utilized data from the Surgical Gastric Cancer Patient Registry of West China Hospital (WCH-SGCPR), under registry number WCHSGCPR-2021. The Research Ethics Committee of West China Hospital approved the establishment of this database (Approval No. 2014-215; date: 10 December 2014). All medical records were anonymized and de-identified prior to analysis, and informed consent was obtained from all patients prior to undergoing surgery.

### 2.2. Patients

This study analyzed the clinicopathological characteristics of 5493 consecutive primary gastric patients who underwent surgical treatment in the Department of Gastrointestinal Surgery, West China Hospital Sichuan University, between 1 January 2009, and 31 December 2018.

The inclusion criteria were as follows: (1) histologically confirmed gastric adenocarcinoma; (2) complete preoperative laboratory test data, including routine blood test, blood biochemistry test, and blood coagulation profiles. Patients were excluded if they met any of the following criteria: (1) had a history of other malignancies; (2) were diagnosed with remnant gastric cancer; (3) received preoperative chemotherapy and/or radiotherapy; (4) patients without complete follow-up information; or (5) underwent non-radical surgery or presented with distant metastasis.

**Figure 1:**
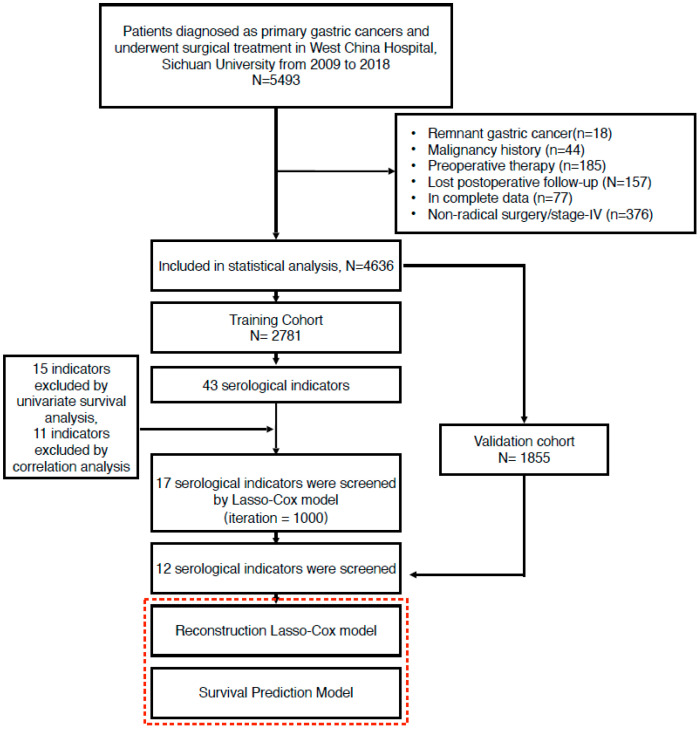
Flow chart of patient selection procedures.

Ultimately, 4636 patients met the inclusion criteria and were included in the final analysis. These patients were randomly divided into a training cohort (2781 patients) and a validation cohort (1855 patients) at a 6:4 ratio. Details of the patient selection process are provided in Figure 1.

### 2.3. Clinicopathological Materials

This study examined a range of potential predictive factors, including age (years), sex (male/female), tumor size (cm), tumor location (adenocarcinoma of the esophagogastric junction [AEG] vs. non-AEG), macroscopic classification (Type 0/I/II/III/IV), differentiation degree (well/moderate/poor), pathological TNM (pTNM) stages, and serological indicators obtained from routine blood test, biochemical analyses, and coagulation tests. Tumor differentiation and pTNM staging were determined according to the eighth edition of the American Joint Committee on Cancer (AJCC) TNM staging manual [18]. The preoperative serological indicators included hemoglobin (HGB), platelets (PLT), lymphocyte count (LYMPH), neutrophil count (NEUT), albumin (ALB), alkaline phosphatase (ALP), creatinine (CREA), high-density lipoprotein cholesterol (HDL-C), lactate dehydrogenase (LDH), and fibrinogen (FIB). A comprehensive list of all 43 non-tumor serological markers, including their full names, abbreviations, and normal reference ranges, is provided in Supplementary Table S1.

### 2.4. Surgical Treatment

All gastric cancer patients without distant metastasis underwent surgery with curative intent. The surgical procedures were performed in accordance with the Gastric Cancer Treatment Guidelines issued by the Japanese Gastric Cancer Association [19,20]. The extent of resection was determined based on tumor location and lymph node status. Routine intraoperative frozen-section pathological examinations were conducted to ensure clear surgical margins.

### 2.5. Follow-Up and Clinical Endpoints

All patients who underwent surgical treatment were encouraged to attend regular outpatient follow-up visits. Supplementary follow-up methods included telephone interviews, online communication tools, and postal correspondence. The follow-up schedule consisted of visits every 3 to 6 months during the first 2 years after gastrectomy, every 6 to 12 months for the subsequent 3 years, and annually thereafter until death. The primary reasons for the loss to follow-up were patients’ inability to return to the outpatient clinic or inaccessible contact information. Follow-up information was updated to 1 January 1 2024. Among the 5493 patients, only 157 were lost to follow-up, resulting in a follow-up rate of 97.1%. The median follow-up duration of the 4636 analyzed patients was 79.1 months (interquartile range: 40.1–109.9 months), with a median of 80.1 months (IQR: 42.0–110.2) in the training cohort and 77.7 months (IQR: 36.4–109.0) in the validation cohort.

### 2.6. Statistical Analysis

Before conducting statistical analysis, the normality of data was assessed. Quantitative data are presented as the mean ± standard deviation. Continuous variables following a normal distribution were compared using Student’s *t*-test, whereas categorical variables were analyzed using the two-tailed chi-square test or Fisher’s exact test. For non-normally distributed and ordinal variables, the Mann–Whitney *U* test was employed. Overall survival outcomes were estimated using the Kaplan–Meier method and compared with the log-rank test.

Patients were randomly divided into a training cohort (2781 patients) and a validation cohort (1855 patients) in a 6:4 ratio. Serological indicators were screened using a three-step process before finalizing the LASSO-Cox regression model: (1) serological indicators that showed no significant association with survival in univariate analysis in the training cohort were excluded (Supplementary Table S2); (2) indicators were subjected to LASSO-Cox regression with 1000 iterations, and those retained in more than 900 iterations were included for further analysis; and (3) correlation analysis was performed, and indicators with a correlation coefficient greater than 0.7 were excluded (Supplementary Table S3). Ultimately, 12 serological indicators were selected to establish the final LASSO-Cox regression model and generate the prediction score, termed the serum risk score (SerScore).

Patients were categorized into high-risk and low-risk groups based on a cut-off value determined using the “survminer” package (version 0.4.9) in R. Cox proportional hazards regression was employed for univariate and multivariate survival analyses, with results presented as hazard ratios (HRs) and 95% confidence intervals (CIs). The LASSO-Cox regression model was performed using the “glmnet” package (version 4.1.8). A multivariate survival model was developed into a nomogram using the “rms” package (version 6.8.0) and the “nomogramFormula” package (version 1.2.0.0) in R. The nomogram’s performance was evaluated using the concordance index (C-index) and the area under the curve (AUC). ROC curve analysis was conducted and visualized using the “survivalROC” package (version 1.0.31) to predict the 5-year overall survival.

The clinical utility of the nomogram was assessed using decision curve analysis (DCA) with the “ggDCA” package (version 1.2) in R. Calibration performance was evaluated by estimating the calibration slope from a Cox proportional hazards model fitted to the predicted linear predictor, with an ideal value of 1 indicating perfect calibration. The overall predictive accuracy of the nomogram was quantified using the integrated Brier score (IBS), which was calculated with the “pec” package (version 2023.4.12) based on 1000 bootstrap resamples and the “632+” method. A two-tailed *p* value < 0.05 was considered statistically significant. All statistical analyses were performed using the R software (version 4.0.2).

## 3. Results

### 3.1. Clinicopathological Characteristics

Clinical and pathological data from 4636 gastric cancer patients who underwent surgical treatment were screened and analyzed in the final analysis (Figure 1). Among these, 2781 patients were randomized into the training cohort and 1855 into the validation cohort at a 6:4 ratio. The general clinicopathological characteristics were comparable between the training and validation cohorts, with no significant differences observed (Table 1). Furthermore, the distribution of the 43 serological indicators between the two cohorts is summarized in Supplementary Table S1.

### 3.2. Screening Serological Indicators and Establishment of the Serscore

**Figure 2:**
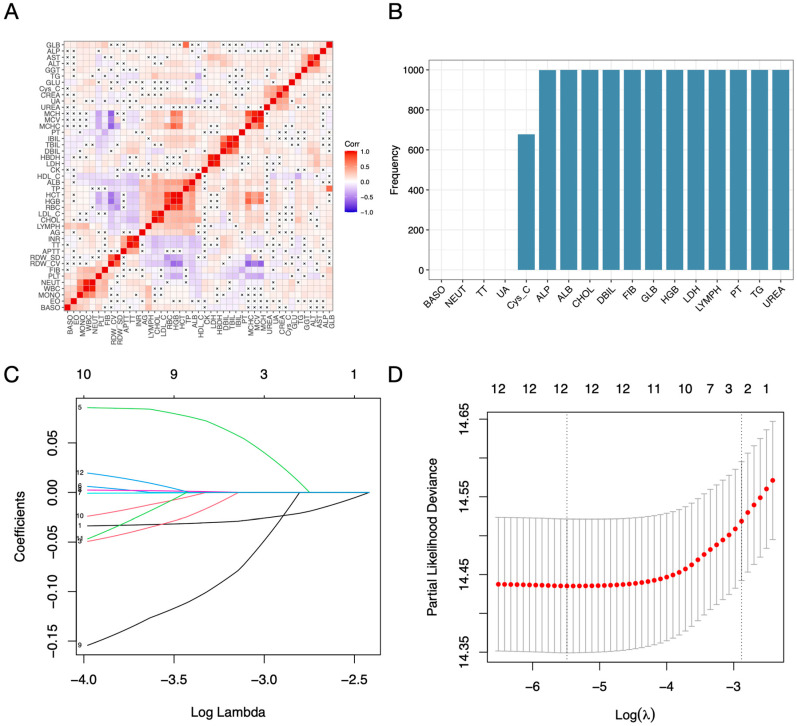
Screening of serological indicators using the least absolute shrinkage and selection operator (LASSO) Cox regression model. (**A**) Correlation analysis of 43 serological indicators. (**B**) Screening of 43 serological indicators using LASSO-Cox regression with 1000 iterations. (**C**,**D**) Selected serological indicators included in the final LASSO-Cox regression model.

A total of 43 serum markers were initially screened using univariate survival analysis, resulting in the exclusion of 15 serum indicators (Supplementary Table S2). Correlation analysis subsequently excluded an additional 11 indicators (Figure 2A, Supplementary Table S3). The remaining 17 serological indicators were subjected to LASSO-Cox regression in the training cohort. Random LASSO-Cox regression, performed over 1000 iterations, identified 12 serological indicators (aspartate aminotransferase, ALP; albumin, ALB; cholesterol, CHOL; direct bilirubin, DBIL; fibrinogen, FIB; total serum globulin, GLB; hemoglobin, HGB; lactate dehydrogenase, LDH; lymphocyte count, LYMPH; prothrombin time, PT; triglyceride, TG; and urea, UREA) that were retained in more than 900 iterations (Figure 2B). These 12 indicators were then used to construct the final LASSO-Cox model. The adjustment parameter (λ) in the LASSO-Cox model was optimized using 10-fold cross-validation. The relationship between the partial likelihood deviation curve and the logarithm (λ) was plotted, with the optimal λ values identified at 0.00413 for the minimum standard and 0.05594 for the 1-SE standard. The LASSO coefficient curves of the 12 selected indicators were visualized (Figure 2C), showing 12 coefficients at the minimum standard and only one coefficient (ALB) at the 1-SE standard (Figure 2D). These 12 serological indicators were subsequently utilized to calculate the serum risk score (SerScore) for individual patients in both the training and validation cohorts.

### 3.3. Survival Analysis and Prognostic Model Validation

**Figure 3:**
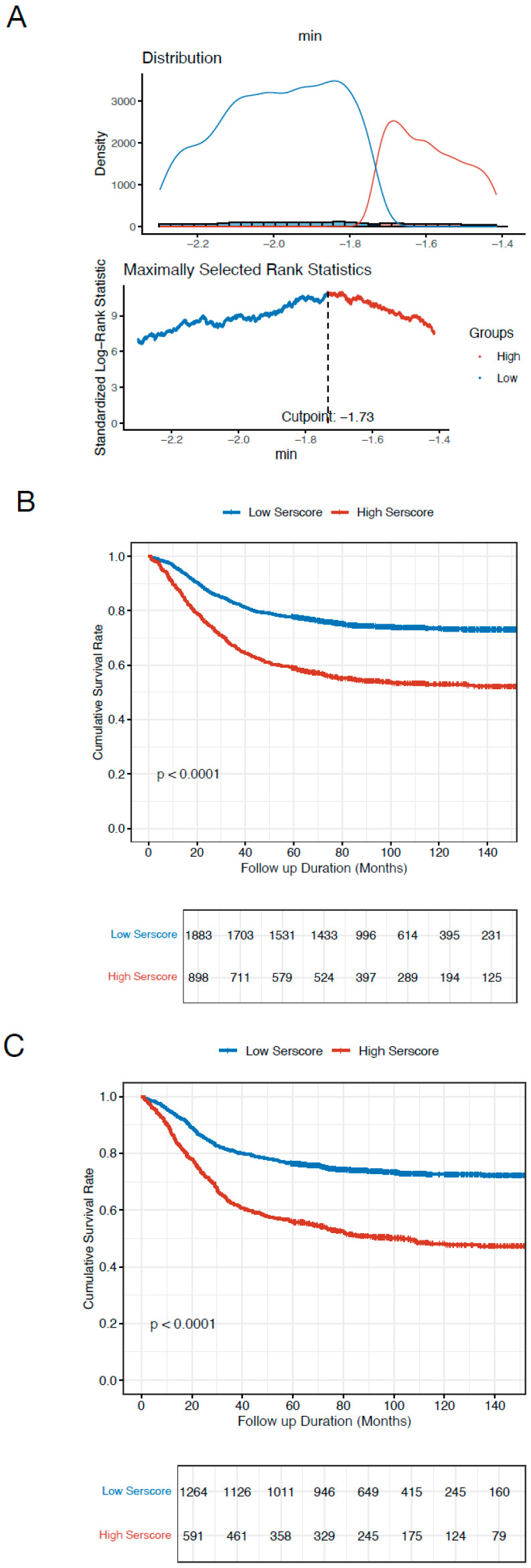
Survival validation of the SerScore in the training and validation cohorts. (**A**) Determination of the SerScore cut-off value in the training cohort. (**B**) Survival curves comparing high- and low-SerScore groups in the training cohort. (**C**) Survival curves comparing high- and low-SerScore groups in the validation cohort.

**Figure 4:**
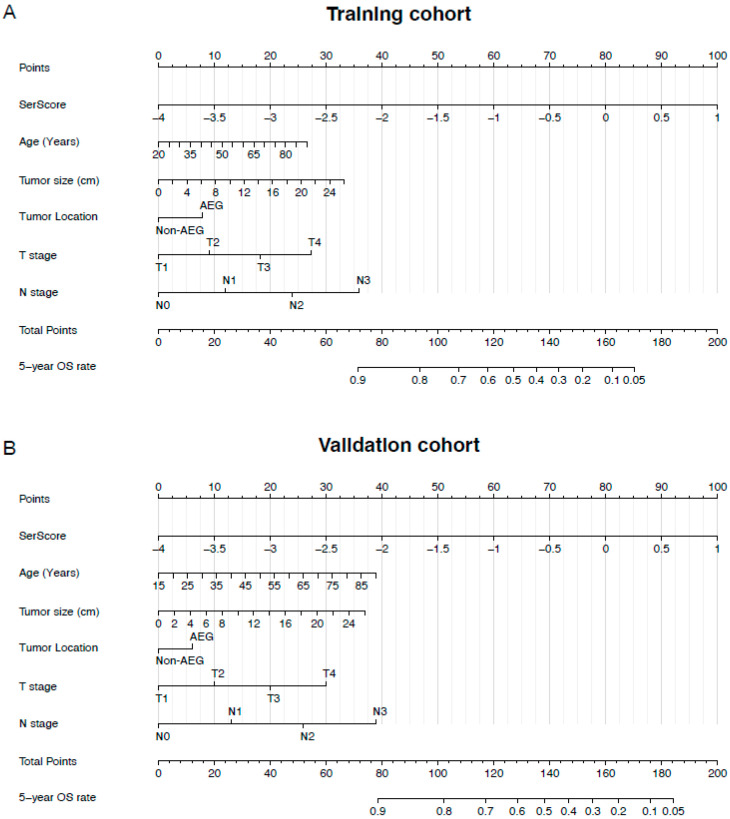
Nomograms for survival prediction in gastric cancer patients. (**A**) Nomogram for the training cohort. (**B**) Nomogram for the validation cohort.

**Figure 5:**
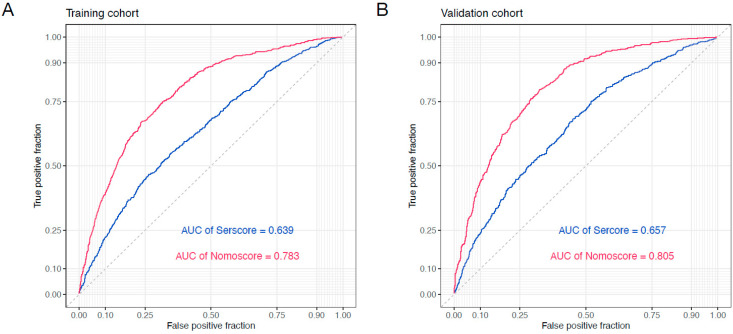
Receiver operating characteristic (ROC) curves for the SerScore and nomogram models. (**A**) ROC curves for the SerScore and nomogram models in the training cohort. (**B**) ROC curves for the SerScore and nomogram models in the validation cohort.

Based on the cut-off value of −1.73 for the SerScore, patients were categorized into a high and low SerScore groups using maximally selected rank statistics (Figure 3A). Patients in the low SerScore group exhibited significantly better overall survival outcomes compared to those in the high SerScore group in both the training and validation cohorts (Figure 3B,C). Univariate and multivariate Cox proportional hazard analyses were conducted to identify independent prognostic factors in both cohorts. In the training cohort, the SerScore (HR = 2.080, 95% CI: 1.724–2.509, *p* < 0.001), age (HR = 1.012, 95% CI: 1.006–1.019, *p* < 0.001), tumor location (non-AEG vs. AEG, HR = 0.754, 95% CI: 0.648–0.877, *p* < 0.001), T stage (T3 vs. T1 stages, HR = 1.637, 95% CI: 1.195–2.243, *p* < 0.001; T4 vs. T1 stages, HR = 2.732, 95% CI: 2.004–3.726, *p* < 0.001), and N stage (N1 vs. N0 stages, HR = 1.491, 95% CI: 1.157–1.922, *p* = 0.002; N2 vs. N0 stages, HR = 2.034, 95% CI: 1.599–2.586, *p* < 0.001; N3 vs. N1, HR = 3.470, 95% CI: 2.776–4.338, *p* < 0.001) were identified as independent prognostics factors for overall survival (Table 2). Similar results were observed in the validation cohort (Table 3). Using the independent prognostic factors identified by multivariate analysis, multivariate nomograms were constructed for both the training and validation cohorts (Figure 4A,B). The nomogram’s C-index was 0.745 (0.729–0.760) in the training cohort and 0.750 (0.733–0.768) in the validation cohort. The SerScore and nomogram models were evaluated using ROC curves. The multivariate nomogram, which combined the SerScore with clinical prognostic factors, demonstrated superior prediction compared to the SerScore in the training cohort (AUC: 0.783 vs. 0.639) and the validation cohort (AUC: 0.805 vs. 0.657) (Figure 5A,B).

**Figure 6:**
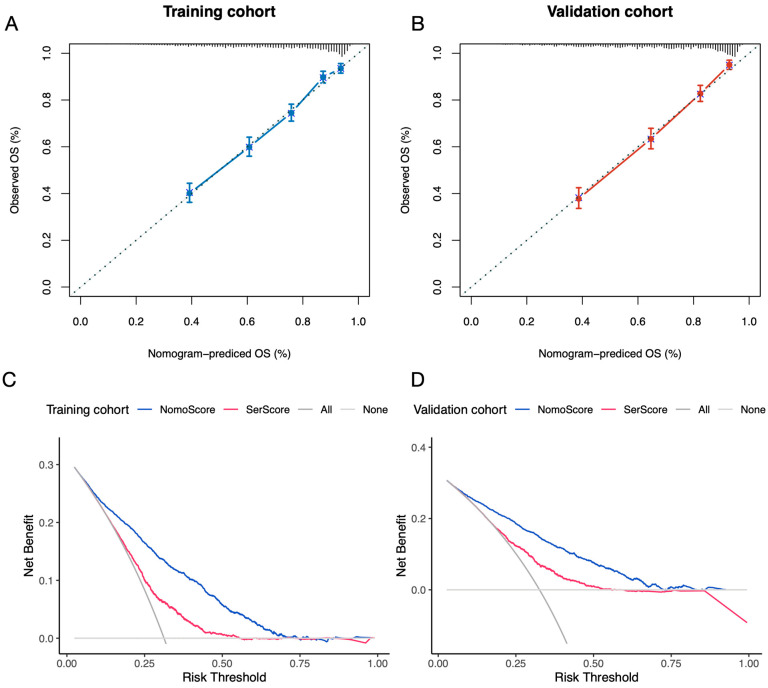
Calibration plots and decision curve analysis (DCA) for the nomogram models. (**A**) Calibration plot for the nomogram in the training cohort. (**B**) Calibration plot for the nomogram in the validation cohort. (**C**) DCA for survival outcomes (SerScore and nomogram) in the training cohort. (**D**) DCA for survival outcomes (SerScore and nomogram) in the validation cohort.

Calibration curves of the nomogram demonstrated good agreement between the predicted and observed survival probabilities in both the training and validation cohorts, with calibration slopes of 1.000012 and 1.000017, respectively, and intercepts close to zero (Figure 6A,B). The integrated Brier score was 0.190 in the training cohort and 0.197 in the validation cohort, indicating satisfactory overall prediction accuracy. Additionally, decision curve analysis (DCA) was performed for both the SerScore and the nomogram model to evaluate their net clinical benefits. The DCA curves showed that the nomogram consistently provided a greater net benefit across a wide range of threshold probabilities compared to the SerScore alone in both the training and validation cohorts (Figure 6C,D).

## 4. Discussion

The diagnostic sensitivity and specificity of commonly used clinical serum tumor markers—including CEA, CA72-4, CA12-5, and CA19-9—remain limited due to the absence of gastric cancer-specific serum markers [5,6,7,8]. Previous studies have explored the use of preoperative inflammatory, nutrition, and coagulation markers to predict chemotherapy sensitivity and survival outcomes in gastric cancer patients. However, these studies did not comprehensively analyze all preoperative serological indicators, thereby restricting the diagnostic and prognostic value of the serum tumor markers. In this study, we systematically screened preoperative serological indicators from routine blood tests, biochemical analyses, and coagulation profiles and conducted a comprehensive analysis of their association with survival outcomes in a large cohort of gastric cancer patients. We developed a serum risk score (SerScore) and confirmed its prognostic significance. The SerScore was identified as an independent prognostic factor for overall survival in both the training and validation cohorts using multivariate Cox proportional analysis. Furthermore, our multivariate survival model demonstrated robust predictive performance across both cohorts.

Hemoglobin and albumin levels are frequently reduced in gastric cancer patients, often approaching the lower limit of the reference range, particularly in those with advanced-stage disease. Secondary relative malnutrition and chronic gastrointestinal bleeding caused by malignant tumors contribute to chronic anemia, preventing hemoglobin and albumin levels from reaching normal values. Hypoalbuminemia and anemia are generally indicative of advanced primary gastric cancer and are associated with a poor prognosis [12,21].

Cholesterol homeostasis plays a critical role in cancer progression [22]. Studies have demonstrated that HDL-C levels are significantly reduced in the peripheral blood of gastric cancer patients, with low HDL-C levels strongly associated with advanced N stages [23]. The role of the HDL receptor in reverse cholesterol transport is crucial in preventing intracellular cholesterol accumulation during tumor development [23,24]. Moreover, *Helicobacter pylori* infection—a major risk factor for gastric cancer present in more than half of gastric cancer patients [25,26]—is associated with dyslipidemia, characterized by increased LDL-C and TC levels and decreased negative HDL-C and TG levels [27]. This suggests that cholesterol homeostasis may be influenced by both *H. pylori* infection and the presence of primary gastric cancer. It is also noteworthy that serum nutrition and metabolism markers can vary postoperatively depending on the type of resection and reconstruction method used in gastric cancer patients. As such, preoperative serum examination provides a more accurate reflection of tumor status and associated metabolic alterations.

Immune cell infiltration within tumor cells or the tumor immune microenvironment is significantly associated with survival outcomes and chemotherapy sensitivity in gastric cancer patients [28,29]. Peripheral blood neutrophil, monocyte, and lymphocyte counts provide a general reflection of the body’s immune function. Preoperative metrics such as the neutrophil-to-lymphocyte ratio (NLR), systemic inflammation response index (SIRI), and systemic immune-inflammation index (SII) are commonly utilized to predict patient survival outcomes [30,31]. Consistent with previous studies, neutrophil and lymphocyte counts were included as variables in the SerScore model and were significantly associated with overall survival outcomes. Additionally, evidence suggests that peripheral blood immune cells interact with circulating tumor cells, facilitating tumor cell proliferation, dissemination, and progression [32,33]. The complex interactions among peripheral blood immune cells, tumor-infiltrating lymphocytes, and tumor cells vary across pathological subtypes, tumor stages, and molecular characteristics. These interactions influence tumorigenesis, progression, and regression by modulating the dynamic balance between immune surveillance and immune escape.

Hypercoagulability is a hallmark of malignancies, driven by the tumor cells’ activation of the coagulation system [34]. Fibrinogen- or platelet-related indices have been shown to correlate closely with tumor progression and survival outcomes [35,36,37]. Furthermore, variations in glucose, alkaline phosphatase, lactate dehydrogenase, and creatinine levels are significantly associated with differences in patient prognoses. These changes in serological indicators are likely influenced by signaling pathways that regulate tumor cell differentiation and behavior [38,39,40].

In this study, we developed the SerScore by screening preoperative serological indicators and analyzing their correlation with patient prognosis. The SerScore was integrated with clinicopathological characteristics, including tumor location, tumor size, and tumor stage, to establish and validate a comprehensive prognostic model. Our objective was not to replace the TNM staging system or other pathological markers but to augment the prognostic efficacy of the TNM staging system. By combining the SerScore with clinical indicators, this approach provides a more comprehensive and nuanced assessment of gastric cancer prognosis.

Despite the strengths of this study, several limitations should be acknowledged. First, this was a retrospective single-center analysis. Although the sample size was relatively large, it may still be insufficient to fully validate the robustness of the model. External validation was not feasible due to data accessibility constraints, but it remains critical for confirming generalizability. Future multicenter studies with larger and more diverse populations are needed to enhance the model’s stability and applicability. Second, the optimal cut-off value for the SerScore was determined using maximally selected rank statistics (MaxStat), which may inflate type I error rates, exaggerate hazard ratios, and introduce internal circularity given that the cut-off was both derived and validated within the same cohort. Although this approach was used primarily for exploratory purposes, further validation in independent external cohorts is necessary to confirm its robustness. Third, during variable selection, markers that were not significant in univariate survival analysis or exhibited high correlation (r > 0.7) were excluded to minimize redundancy and overfitting. Although this approach reduces model complexity, it may have inadvertently omitted correlated but jointly informative predictors. For instance, low-density lipoprotein cholesterol (LDL-C) and mean corpuscular volume (MCV), previously reported as prognostic factors in gastric cancer [41,42], were not included in the final model. Future studies could explore alternative statistical approaches, such as Elastic-Net Cox regression or machine learning algorithms, to integrate a broader range of predictive markers without relying on strict pre-filtering criteria.

Despite these limitations, this study provides a valuable prognostic framework, highlighting the significance of serological indicators in survival prediction and offering a clinically applicable tool for risk stratification in gastric cancer. Further validation with larger, multicenter datasets and more advanced statistical methodologies will be essential to enhance the model’s reliability and clinical utility.

## 5. Conclusions

The SerScore model developed in this study incorporates 12 weighted serological indicators specific to primary gastric cancer patients. This model offers an effective and reliable tool for prognostic evaluation and establishes a foundation for the development of more advanced prognostic models for gastric cancers.

## Figures and Tables

**Table 1:** General characteristics of patients in the training and validation cohorts.

Characteristics		All Patients	Training Cohort	Validation Cohort	*p* Value
		N = 4636 (%)	N = 2781 (%)	N = 1855 (%)	
Age	Years	58.3 ± 11.5	58.2 ± 11.4	58.4 ± 11.5	0.460
Gender	Male	3212 (69.3)	1922 (69.1)	1290 (69.5)	0.781
	Female	1424 (30.7)	859 (30.9)	565 (30.5)	
Tumor location	AEG	1001 (21.6)	610 (21.9)	391 (21.1)	0.511
	Non-AEG	3635 (78.4)	2171 (78.1)	1464 (78.9)	
Tumor size	cm	4.7 ± 2.6	4.7 ± 2.6	4.60 ± 2.6	0.256
Degree of differentiation	G1	79 (1.7)	50 (1.8)	29 (1.6)	0.512
	G2	868 (18.7)	533 (19.2)	335 (18.1)	
	G3	3689 (79.6)	2198 (79.0)	1491 (80.4)	
Macroscopic type	0	1118 (24.1)	647 (23.3)	471 (25.4)	0.151
	I	69 (1.5)	48 (1.7)	21 (1.1)	
	II	1420 (30.6)	871 (31.3)	549 (29.6)	
	III	1847 (39.8)	1100 (39.6)	747 (40.3)	
	IV	182 (3.9)	115 (4.1)	67 (3.6)	
T stage	T1	1118 (24.1)	647 (23.3)	471 (25.4)	0.283
	T2	728 (15.7)	452 (16.3)	276 (14.9)	
	T3	1081 (23.3)	645 (23.2)	436 (23.5)	
	T4	1709 (36.9)	1037 (37.3)	672 (36.2)	
N stage	N0	1684 (36.3)	1008 (36.2)	676 (36.4)	0.619
	N1	769 (16.6)	477 (17.2)	292 (15.7)	
	N2	835 (18.0)	499 (17.9)	336 (18.1)	
	N3	1348 (29.1)	797 (28.7)	551 (29.7)	
TNM stage	I	1326 (28.6)	793 (28.5)	533 (28.7)	0.906
	II	1123 (24.2)	680 (24.5)	443 (23.9)	
	III	2187 (47.2)	1308 (47.0)	879 (47.4)	
Adjuvant chemotherapy	No	2102 (45.3)	1232 (44.3)	870 (46.9)	0.087
	Yes	2534 (54.7)	1549 (55.7)	985 (53.1)	

Abbreviations: AEG, adenocarcinoma of the esophagogastric junction.

**Table 2:** Univariate and multivariate survival analysis of patients in the training cohort.

Characteristics		Univariate			Multivariate		
		HR	95% CI	*p* Value	HR	95% CI	*p* Value
SerScore		2.914	2.487–3.415	<0.001	2.080	1.724–2.509	<0.001
Age	Years	1.018	1.012–1.024	<0.001	1.012	1.006–1.019	<0.001
Gender	Female vs. Male	0.905	0.783–1.045	0.173	0.919	0.792–1.066	0.266
Tumor size	≥5 cm vs. <5 cm	2.707	2.356–3.111	<0.001	1.043	0.883–1.232	0.617
Tumor location	Non-AEG vs. AEG	0.669	0.578–0.774	<0.001	0.754	0.648–0.877	<0.001
Degree of differentiation	G3 vs. G1-G2	1.577	1.316–1.890	<0.001	1.153	0.956–1.390	0.137
Macroscopic type	Type III–IV vs. Type 0–II	1.550	1.359–1.768	<0.001	0.908	0.789–1.044	0.174
T stage	T2 vs. T1	1.789	1.303–2.457	<0.001	1.309	0.940–1.824	0.111
	T3 vs. T1	3.163	2.408–4.155	<0.001	1.637	1.195–2.243	0.002
	T4 vs. T1	6.508	5.072–8.349	<0.001	2.732	2.004–3.726	<0.001
N stage	N1 vs. N0	1.914	1.500–2.443	<0.001	1.491	1.157–1.922	0.002
	N2 vs. N0	2.988	2.397–3.726	<0.001	2.034	1.599–2.586	<0.001
	N3 vs. N0	5.856	4.844–7.078	<0.001	3.470	2.776–4.338	<0.001
Adjuvant chemotherapy	No vs. Yes	1.076	0.942–1.228	0.281	0.903	0.789–1.034	0.141

Abbreviations: HR, hazard ratio; CI, confidence interval; AEG, adenocarcinoma of the esophagogastric junction.

**Table 3:** Univariate and multivariate survival analysis of patients in the validation cohort.

Characteristics		Univariate			Multivariate		
		HR	95% CI	*p* Value	HR	95% CI	*p* Value
SerScore		2.917	2.439–3.489	<0.001	1.877	1.491–2.362	<0.001
Age	Years	1.023	1.015–1.030	<0.001	1.017	1.010–1.025	<0.001
Gender	Female vs. Male	0.857	0.720–1.021	0.084	0.954	0.797–1.142	0.608
Tumor size	≥5 cm vs. <5 cm	3.259	2.752–3.859	<0.001	1.295	1.059–1.585	0.012
Tumor location	Non-AEG vs. AEG	0.584	0.492–0.693	<0.001	0.800	0.669–0.956	0.014
Degree of differentiation	G3 vs. G1-G2	1.345	1.089–1.661	0.006	1.027	0.826–1.278	0.809
Macroscopic type	Type III–IV vs. Type 0–II	1.736	1.484–2.031	<0.001	0.885	0.749–1.047	0.154
T stage	T2 vs. T1	2.729	1.844–4.037	<0.001	2.085	1.382–3.145	<0.001
	T3 vs. T1	4.660	3.311–6.559	<0.001	2.593	1.755–3.831	<0.001
	T4 vs. T1	8.801	6.395–12.112	<0.001	3.344	2.259–4.950	<0.001
N stage	N1 vs. N0	1.930	1.442–2.584	<0.001	1.248	0.923–1.688	0.150
	N2 vs. N0	2.477	1.895–3.238	<0.001	1.371	1.030–1.826	0.031
	N3 vs. N0	5.941	4.755–7.422	<0.001	3.175	2.458–4.102	<0.001
Adjuvant chemotherapy	No vs. Yes	1.302	1.111–1.527	<0.001	1.082	0.920–1.271	0.341

Abbreviations: HR, hazard ratio; CI, confidence interval; AEG, adenocarcinoma of the esophagogastric junction.

## Data Availability

The original contributions presented in this study are included in the article/Supplementary Materials. Further inquiries can be directed to the corresponding author.

## Supplementary Materials

The following supporting information can be downloaded at https://www.mdpi.com/article/10.3390/jcm14124043/s1, Table S1. Serological markers between training and validation cohort in this study; Table S2. Univariate survival analysis of serological markers in training cohort in this study; Table S3. Coefficient value among the 43 serological indicators.

## Abbreviations

The following abbreviations are used in this manuscript:

AEG	adenocarcinoma of the esophagogastric junction
HR	hazard ratio
CI	confidence interval
C-index	concordance index
AUC	area under curve
